# Supplementary material for: Proteomic similarity of the Littorinid snails in the evolutionary context
Source: PeerJ. 2020 Feb 13;8:e8546. doi: 10.7717/peerj.8546 (PMC7024583; doi:10.7717/peerj.8546)
Supplement: Supplemental Information 4 [file peerj-08-8546-s004.pdf]

# Proteomic similarity of the Littorinid snails in the evolutionary context.

Maltseva, A.L.[1], Varfolomeeva, M.A.[1], Lobov, A.L.[1,2], Tikanova, P.O.[1], Panova, M.[3], Mikhailova, N.A.[1,4], & Granovitch, A.I.[1] (2019).

[1] Department of Invertebrate Zoology, St Petersburg State University, St Petersburg, Russia
[2] Laboratory of Regenerative Biomedicine, Institute of Cytology Russian Academy of Sciences, St Petersburg, Russia
[3] Department of Marine Sciences, University of Gothenburg, Strömstad, Sweden
[4] Centre of Cell Technologies, Institute of Cytology Russian Academy of Sciences, St Petersburg, Russia

Corresponding author:

Arina Maltseva
Universitetskaya 7/9A, St Petersburg, 199034, Russia
arina.maltseva@spbu.ru;

## Supplement 1 File. Methods details.

**Littorina (Neritrema) species identification.** Three species of the "saxatilis"-group (*L. compressa*, *L. arcana* and *L. saxatilis*) are morphologically very close. Females can be distinguished by their reproductive system: females of *L. compressa* and *L. arcana* are both oviparous, whereas *L. saxatilis* is ovoviviparous. The identification of females was based on the morphology of the distal part reproductive system, namely, a relative size of the albumen, capsule and jelly glands, as well as size and shape of the bursa copulatrix (broad and long in *L. arcana* vs short and slim in *L. compressa*). Species identification of males is based on penis morphology [1,2]: two or more rows of small-size numerous mamilliform penial glands and triangular filament for *L. arcana*; one row of distal large-size glands (no more than 6 in number) and short filament for *L. compressa*; one row of small-size numerous (more than 6 in number) mamilliform penial glands and triangular filament for *L. saxatilis*. While males of *L. compressa* are easily recognizable, males of *L. arcana* and *L. saxatilis* are often hard to distinguish from each other and penial characteristics were shown to vary considerably within both species [3].

Two species of the "obtusata"-group (*L. obtusata* and *L. fabalis*) are both oviparous and possess similar shell form and texture. Females were identified due to differences in bursa copulatrix morphology: long (reaching up the albumen gland) in *L. obtusata* vs short (faint and hardly visible) in *L. fabalis*. Males differ in mamilliform gland number and form and penial filament morphology. Penis of *L. fabalis* carries no more than 6 relatively large glands, placed in the middle part of the organ; the filament is thin and long (may be longer than basal penis part). Males with several rows of small glands on a penis with short and thick filament were qualified as *L. obtusata* [2].

**Rationale of the proteomic - genetic trees comparison.** Proteomics is widely used for elucidation of molecular mechanisms underlying dynamic physiological processes, like acclimation, stress, immune response, disease, etc. Further, proteomic represent an informative tool for studies of ecology, population biology, and evolutionary physiology of species [4-8]. Finally, proteomic comparisons were successfully applied for resolution of taxonomical conundrums in diverse organisms from viruses and bacteria to metazoans [9-14]. Principal distinctive feature of proteomics compared to transcriptomics and genomics is dealing with expressed traits.

There are two principal proteomic approaches depending on fundamental separation method: gel-based (implementing 2 dimensional electrophoresis, 2DE) and gel-free (relying on liquid chromatography, HPLC). Any proteomic study of a living system (cell, tissue, organ, organism) is incomplete in terms of a set of identified proteins due to high diversity of protein structures (e.g. solubility, means, accessibility for analysis; sensibility to protease action, means, feasibility of their MS-identification, etc.), enormous range of their abundances and complex spatio-temporal distribution [15,16]. This is fair for both gel-based and gel-free proteomic analytical strategies. For example, only ~34% of expected proteins were detected while mapping of human proteome by LC-MS-based methodology [17]; only ~ 25% of predicted for expression proteins were visualized by 2D-gel-based approach in a yeast model [18,19]. In the present study we chose the gel-based strategy (with implementation of 2D-DIGE) due to a number of advantages: multiplexity, reproducibility, high sensitivity, opportunity to detect different post-translations modifications and their combinations, simple and fast sample preparation, and compatibility with MS-identification [20,21]. All the samples were subjected to the same procedures to make obtained data comparable; qualitative data on all reliably detected protein spots were included into analysis.

The Jaccard index is a pairwise dissimilarity measure based on shared and distinct proteins in the two samples. Generally, even single amino acid substitution or modification affects protein pI and its position in the gel, creating a new spot. Jaccard index accounts for all spots present in at least one of the two compared samples; therefore, it is robust when a sample lacks a particular spot for different reasons (absence of expression or expression below the detection level) [22-24]. In theory, this method cannot detect the situations when the same spot is present in the two samples due to different substitutions/modifications. However, such cases are rather unlikely.

Thus, we are detecting all events of protein changes but do not account for the degree of change. This resembles a morphological approach, considering similarity of features but not accounting for underlying molecular mechanisms. Our approach is also similar to DNA fingerprinting methods, such as AFLP, RFLP, RAPD [25], with the main difference of exclusively engaging a working part of cellular molecular machinery. Such sequence-independent proteomic fingerprinting method is advantageous in several aspects. First of all, it is applicable to any organisms, including poorly characterized or completely uncharacterized at the molecular level. Secondly, it deals with expressed molecular traits, which may be under selection, and there is no prerequisite of evolutionary neutrality in proteomic fingerprinting analysis. In the contrast, DNA phylogenetic algorithms implicitly assume the neutrality of substitutions, and selection violates the assumptions of phylogenetic inference [see e.g. 26-29].

For the Bayesian phylogenetic analysis, we used publicly available sequences of presumably neutral molecular markers (see [30] and details below): two of which are non-protein coding genes (18S rRNA and 12S rRNA). The phylogeny inferred by neutral markers reflects neutral random genome drift through evolutionary process [31-33]. Nevertheless, the evolution is supposed to be adaptive, and obviously neutral markers cannot tell whole evolutionary history of any species. It is generally accepted now, that multilocus analysis is reasonable due to sampling sites with varying mutation rate, which makes it possible to resolve both deep and shallow noes in the phylogenetic tree [34]. Actually, our approach was inspired with the idea of inconsistency between gene trees and species trees [rev. *e.g.* in 34-36]. The background of this inconsistency is provided by such diverse issues as gene transfer and duplication, deep coalescence and branch length heterogeneity. Considering effect of these phenomena in phylogenetic analysis is expected to outline more adequately an evolutionary history of a species genome. Nevertheless, "the diversity recovered in our surveys of DNA sequence evolution within and between species is ultimately an indirect and incomplete window into the history of species" due the different level of complexity of such entities as genes or genomes *vs* organisms, population and species [34]. We do not layup claim of inferring true species tree by this quantitative-proteomic approach (though we suspect that proteome-based trees could be more

congruent with whole-genome trees). We suggest that comparison of trees, inferred from neutral slowly drifting part of genome *vs* proteome-based scan (including signals from fast evolving proteins) can elucidate some aspects of a species recent evolutionary history.

**2D DIGE Electrophoresis and analysis.** At least 2 technical replicates (with swap in Cy-dyes conjugation pattern) were done for every sample; the replicates were averaged. Before the first direction of electrophoretic separation samples were conjugated with fluorophores Cy2, Cy3 or Cy5 (BioDye) in proportion 400 pmol of Cy per 50 µg of total protein. The approximate total protein concentration in a sample was estimated by the absorbance at 280 nm with NanoDrop® ND-1000 UV-Vis Spectrophotometer (NanoDrop Technologies, USA). Samples mixed with Cy-fluorophores were incubated for 30 min on ice at dark, and then the reaction was stopped by addition of 10 µmol of L-lysin and further incubation for 10 min under the same conditions. The samples conjugated with different Cy-fluorophores were pooled and loaded into IPG ReadyStrip (7 cm, pH 3-10; BioRad) by passive rehydration (overnight at room temperature, in dark). Separation of the first direction was carried out in Protean IEF Cell (BioRad) using the method, recommended by the manufacturer: 10 000 Vh, end voltage 4 000 V, rapid ramp, 50 µA/strip current limit, 20°C. Before the separation of the second direction focused strips were incubated in two equilibration buffers (6M urea, 2% SDS, 20% glycerin, 0.375M Tris, pH 8.8) for 15 min each: first, with 2% dithiothreitol; second, with 2.5% iodoacetamide for carbamidomethylation. Second direction EF was performed in MiniProtean TetraCell (BioRad) in 15% PAAG in tris/glycine/SDS buffer (BioRad). GE Typhoon 9500 FLA laser scanner was used for separate visualization of particular samples.

**Protein identification.** Protein identification was done in accordance with a "bottom up" approach, which means using tandem mass spectrometry (MS/MS) identification of tryptic peptides with a Database search. For trypsinization, spots of interest were excised from the gel with a scalpel and cut into pieces. Gel pieces were destained with 50% acetonitrile / 25 mM Tris, pH 8.2, dehydrated in 100% acetonitrile and rehydrated in bovine trypsin solution (20 ng/µl / 25 mM Tris, pH 8.2) on ice for 60 min. After rehydration, any excessive trypsin solution was removed, and gel pieces were covered with 25 mM Tris, pH 8.2, and incubated at 37 °C overnight. Tryptic peptides were eluted and analyzed using LC (Agilent 1260) - MS/MS (QTOF UHD 5238, Agilent Technologies, USA). The elution method was 10% B to 60% B for 25 min and further to 100% B for 5 min, where B was 90% acetonitrile / 0.1% formic acid; A - 5% acetonitrile / 0.1% formic acid; flow rate 15 µl/min; and column Zorbax SB-C18 (Agilent Technologies, USA), 5 µm grain, 80 A pores, 150 x 0.5 mm. The mass-spectrometry parameters were as follows: positive mode, auto MS/MS collection, 3 MS-spectra s-1, 3 MS/MS-spectra s-1, precursor mass range 100-3200 Da, precursor isolation window width “medium” (~ 4 m/z), precursor charge 2+, 3+, >3+. An MS/MS search was carried out using Agilent Technologies Spectrum Mill MS Proteomics Workbench Rev B.04.00.127 software against free databases: LSD (http://mbio-serv2.mbioekol.lu.se/Littorina) [37] or SwissProt (ftp://www.expasy.ch/databases/uniprot/); the mode “Identity” (with “carbamidomethylation” as fixed and “S-, T and Y-phosphorylation and M-oxidation” as variable modifications allowed); the precursor mass tolerance +/- 20 ppm; the product mass tolerance +/- 50 ppm. The validation procedure of identified proteins was performed with minimum protein score 20, peptide FDR (False Discovery Rate) for validated proteins: 1%. Qualitative gel analysis was carried out using PDQuest Advanced 8.0.1 software (BioRad, USA). Spots normalization was done using internal program algorithm “on total gel density”. Spots signals under detectable level (with the intensity not exceeding 0 relative to the background) were accepted as absent for qualitative analysis. Spots were accepted as reliably detected if they were detected in at least two technical repeats of the same sample, or in one technical repeat in at least two different samples.

**Phylogenetic analysis** was performed using Bayesian inference (MrBayes 3.2.6 Software [38]) as described in [30]. The following DNA partial sequences of 28S rRNA, 12S rRNA and cytochrome oxidase subunit I (CoI) genes obtained from NCBI were used:

| Species name | 28S GenBank ID | 12S GenBank ID | COI GenBank ID | Substrate and tidal zone | Reproduction and development |
|---|---|---|---|---|---|
| *Littorina obtusata* (L., 1758) | AJ488674 | AJ488756 | AJ622947 | rocky / fucoids lower / middle intertidal | oviparous direct development |
| *Littorina saxatilis* (Olivi, 1792) | HE590811 | HE590783 | HE590840 | rocky / fucoids lower to upper intertidal | ovoviviparous direct development |
| *Littorina littorea* (L., 1758) | AJ488672 | AJ488754 | AJ622946 | rocky / fucoids subtidal and lower / middle intertidal | oviparous planktonic larva |
| *Littorina compressa* Jeffreys, 1865 | HE590806 | HE590777 | HE590834 | rocky / fucoids lower / middle intertidal | oviparous direct development |
| *Littorina fabalis* (W. Turton, 1825) | HE590807 | HE590778 | HE590835 | rocky / fucoids lower intertidal | oviparous direct development |
| *Littorina arcana* Hannaford Ellis, 1978 | HE590804 | HE590775 | HE590832 | rocky / fucoids middle / upper intertidal | oviparous direct development |
| *Echinolittorina marisrubri* Reid, 2007 (as *E. arabica* B) | AJ623224.1 | AJ623130.1 | AJ622980.1 | rocky / sandy middle / upper intertidal | oviparous planktonic larva |
| *Echinolittorina millegrana* (Philippi, 1848) | AJ623261 | AJ623165 | AJ623013 | rocky / sandy middle intertidal | oviparous planktonic larva |
| *Littoraria 'melanostoma* E Asia', unnamed [see 39] | HE590802 | HE590773 | HE590830 | mangrove | ovoviviparous planktonic larva |
| *Littoraria ardouiniana* (Heude, 1885) | FN556263 | FN556477 | FN557076 | mangrove | oviparous planktonic larva |

Fragments of the three genes were used for phylogenetic reconstruction as a concatenated sequence, where individual genes were unlinked to evolve independently. Nucleotide substitution model was GTR+G+I. Analysis was performed as two independent runs, five chains in each (four heated and one cold; the first 25% samples from the cold chain were discarded) for 25 000 000 generations with a sample frequency of 1000, print frequency of 1000 and diagnostics calculated every 1 000 generations. The convergence between two runs was tested by comparison of statistical parameters in the Tracer Software (http://tree.bio.ed.ac.uk/software/tracer/).

**References:**


1 Reid, D.G. (1993) Barnacle-dwelling ecotypes of three British *Littorina* species and the status of *Littorina neglecta* Bean. *J Molluscan Stud* 59(1), 51-62. doi: 10.1093/mollus/59.1.51

2 Reid, D.G. Systematics and Evolution of Littorina. Ray Society: London, UK, 1996

3 Granovitch, A.I.; Loskutova, Z.I.; Gracheva Yu,A.; Mikhailova, N.A. (2008) Morphometric comparison of the copulatory organ in mollusks of "saxatilis" species complex (Caenogastropoda: Littorinidae): problems of identification of species and species status. *Zool Zh* 87(12): 1425-1436. DOI: 10.1007/s10709-013-9728-3

4 Karr, T.L. (2008) Application of proteomics to ecology and population biology. *Heredity* 100(2): 200. doi: 10.1038/sj.hdy.6801008

5 López, J.L. (2007) Applications of proteomics in marine ecology. *MEPS* 332: 275-279. doi: 10.3354/meps332275

6 Biron, D.G., Loxdale, H.D., Ponton, F., Moura, H., Marché L., Brugidou, C., Thomas F. (2006) Population proteomics: an emerging discipline to study metapopulation ecology. *Proteomics* 6(6): 1712-1715. doi: 10.1002/pmic.200500423

7 Diz, A.P., Martínez-Fernández M., Rolán-Alvarez E. (2012) Proteomics in evolutionary ecology: linking the genotype with the phenotype. *Mol Ecol* 21(5): 1060-1080. doi: 10.1111/j.1365-294X.2011.05426.x

8 Baer, B., Millar, A.H. (2016) Proteomics in evolutionary ecology. *J Proteom* 135, 4-11. doi: 10.1016/j.jprot.2015.09.031

9 Kim, G.H., Shim, J.B., Klochkova, T.A., West, J.A., Zuccarello, G.C. (2008) The utility of proteomics in algal taxonomy: *Bostrychia radicans*/*B. moritziana* (Rhodomelaceae, Rhodophyta) as a model study. *J Phycol* 44(6): 1519-1528. doi: 10.1111/j.1529-8817.2008.00592.x

10 Dworzanski, J.P., Snyder A.P. (2005) Classification and identification of bacteria using mass spectrometry-based proteomics. *Expert Rev Proteomic*; 2(6): 863-878. doi: 10.1586/14789450.2.6.863

11 López, J.L. (2005) Role of proteomics in taxonomy: the *Mytilus* complex as a model of study. *J Chromatogr* B 815(1-2): 261-274. doi: 10.1016/j.jchromb.2004.10.064

12 Codoner, F. M., & Elena, S. F. (2006) Evolutionary relationships among members of the Bromoviridae deduced from whole proteome analysis. Archives of virology, 151(2), 299-307. doi: 10.1007/s00705-005-0628-4

13 Rohwer, F., & Edwards, R. (2002) The Phage Proteomic Tree: a genome-based taxonomy for phage. Journal of bacteriology, 184(16), 4529-4535 DOI: 10.1128/JB.184.16.4529-4535.2002

14 Wu, G. A., Jun, S. R., Sims, G. E., & Kim, S. H. (2009) Whole-proteome phylogeny of large dsDNA virus families by an alignment-free method. Proceedings of the National Academy of Sciences, 106(31), 12826-12831 doi: 10.1073/pnas.0905115106

15 Liebler, D.C. Introduction to proteomics: tools for the new biology. Springer Science + Business Media. New York 2002. doi: 10.1007/978-1-59259-130-5

16 Lottspeich, F. Introduction to proteomics. In Proteomics. Methods and Protocols (pp. 3-10). Ed. by Reinders & Sickmann. Humana Press Inc. 2009. DOI: 10.1007/978-1-60761-157-8

17 Kim, M. S., Pinto, S. M., Getnet, D., Nirujogi, R. S., Manda, S. S., Chaerkady, R., ... & Thomas, J. K. (2014) A draft map of the human proteome. *Nature* 509 (7502), 575. DOI10.1038/nature13302

18 Gygi, S. P., Rochon, Y., Franza, B. R., and Aebersold, R. (1999) Correlation between protein and mRNA abundance in yeast. *Mol. Cell Biol.* 19, 1720–1730. DOI: 10.1128/MCB.19.3.1720

19 Gygi, S. P., Corthals, G. L., Zhang, Y., Rochon, Y., and Aebersold, R. (2000) Evaluation of two-dimensional gel electrophoresis-based proteome analysis technology. *Proc Natl Acad Sci USA* 97, 9390–9395. doi: 10.1073/pnas.160270797

20 Berth, M, Moser, FM, Kolbe, M, Bernhardt, J. (2007) The state of the art in the analysis of two-dimensional gel electrophoresis images. *Appl Microbiol Biotechnol* 76(6): 1223-1243. doi: 10.1007/s00253-007-1128-0

21 Arentz, G, Weiland, F, Oehler, MK, Hoffmann, P. (2015) State of the art of 2D DIGE. *Proteomics Clin Appl* 9(3-4): 277-288. doi: 10.1002/prca.201400119

22 Bonin, A., Ehrich, D., Manel, S. (2007) Statistical analysis of amplified fragment length polymorphism data: a toolbox for molecular ecologists and evolutionists. *Mol Ecol* 16: 3737−3758. doi: 10.1111/j.1365-294X.2007.03435.x

23 Duarte, J.M., Santos, J.B.D., Melo, L..C (1999) Comparison of similarity coefficients based on RAPD markers in the common bean. *Genet Mol Biol* 22: 427-432. doi: 10.1590/S1415-47571999000300024

24 Meudt, H.M., Clarke, A.C. (2007) Almost forgotten or latest practice? AFLP applications, analyses and advances. *Trends Plant Sci* 12: 106-117. doi: 10.1016/j.tplants.2007.02.001

25 Blears, M.J., De Grandis, S.A., Lee, H., & Trevors, J.T. (1998) Amplified fragment length polymorphism (AFLP): a review of the procedure and its applications. *Journal of Industrial Microbiol Biotechnol* 21(3), 99-114. doi: 10.1038/sj.jim.2900537

26 Roje, D. M. (2014) Evaluating the effects of non-neutral molecular markers on phylogeny inference. *PloS one* 9(2), e87428. doi: 10.1371/journal.pone.0087428

27 Hagstrom, G.I., Hang, D.H., Ofria, C., Torng, E. (2004) Using Avida to test the effects of natural selection on phylogenetic reconstruction methods. *Artif Life* 10: 157-166. doi: 10.1162/106454604773563586

28 Hang, D., Ofria, C., Schmidt, T.C., Torng, E. (2003) The effect of natural selection on phylogeny algorithms. In: *Genetic and Evolutionary Computation Conference*, Chicago, Il, pp. 13–24.

29 Larmuseau, M.H.D., Huyse, T., Vancampenhout, K., Van Houdt, J.K.J., Volckaert, P.A.M. (2010) High molecular diversity in the rhodopsin gene in closely related goby fishes: A role for visual pigments in adaptive speciation? *Mol Phyl Evol* 55: 689–698. doi: 10.1016/j.ympev.2009.10.007

30 Reid, D.G., Dyal, P., Williams, S.T. (2012) A global molecular phylogeny of 147 periwinkle species (Gastropoda, Littorininae). *Zool Scr* 41(2): 125-136. doi: 10.1111/j.1463-6409.2011.00505.x

31 Kimura, M. (1968) Evolutionary rate at the molecular level. *Nature* 217(5129): 624-626.

32 Felsenstein, J. (1988) Phylogenies from molecular sequences: inference and reliability. *Ann Rev Genetics* 22(1): 521-565. doi: 10.1146/annurev.ge.22.120188.002513

33 Avise, J.C. (2000) Phylogeography: the history and formation of species. Harvard university press.

34 Edwards, S.V. (2009) Is a new and general theory of molecular systematics emerging? *Evolution: International Journal of Organic Evolution*, 63(1): 1-19. doi: 10.1111/j.1558-5646.2008.00549.x

35 Maddison, W.P. (1997) Gene trees in species trees. *Syst Biol* 46: 523-536. doi: 10.1093/sysbio/46.3.523

36 Slowinski, J.B., Page, R.D. (1999) How Should Species Phylogenies Be Inferred from Sequence Data? *Syst. Biol* 48(4): 814-825. DOI:10.1080/106351599260030

37 Canbäck, B., André, C., Galindo, J., Johannesson, K., Johansson, T., Panova, M., et al. (2012) The Littorina sequence database (LSD)–an online resource for genomic data. *Mol Ecol Resour* 12(1): 142-148. doi: 10.1111/j.1755-0998.2011.03042.x

38 Ronquist, F., Huelsenbeck, J.P. (2003) MrBayes 3: Bayesian phylogenetic inference under mixed models. *Bioinformatics* 19(12): 1572-1574. DOI: 10.1093/bioinformatics/btg1803

39 Reid, D.G., Dyal, P. Williams, S.T. (2010) Globaldiversification of mangrove fauna: a molecular phylogeny of *Littoraria* (Gastropoda: Littorinidae). *Mol Phylogenetics Evol* 55: 185–201. doi: 10.1016/j.ympev.2009.09.036
