# Supplementary material for: Proteomic similarity of the Littorinid snails in the evolutionary context
Source: PeerJ. 2020 Feb 13;8:e8546. doi: 10.7717/peerj.8546 (PMC7024583; doi:10.7717/peerj.8546)
Supplement: Supplemental Information 5 [file peerj-08-8546-s005.pdf]

# Proteomic similarity of the Littorinid snails in the evolutionary context.

Maltseva, A.L.[1], Varfolomeeva, M.A.[1], Lobov, A.L.[1,2], Tikanova, P.O.[1], Panova, M.[3], Mikhailova, N.A.[1,4], & Granovitch, A.I.[1] (2019).

[1] Department of Invertebrate Zoology, St Petersburg State University, St Petersburg, Russia
[2] Laboratory of Regenerative Biomedicine, Institute of Cytology Russian Academy of Sciences, St Petersburg, Russia
[3] Department of Marine Sciences, University of Gothenburg, Strömstad, Sweden
[4] Centre of Cell Technologies, Institute of Cytology Russian Academy of Sciences, St Petersburg, Russia

Corresponding author:
Arina Maltseva
Universitetskaya 7/9A, St Petersburg, 199034, Russia
arina.maltseva@spbu.ru;

**Supplement 2 File. Proteomes clustering.**

Proteomic data were analyzed at two levels. First, data on presence/absence of proteins in samples were used to assess proteomic differences related to geographic location, sex and body part. Second, “consensus” species proteomes were constructed to assess the overall similarity of species proteomes. A protein present in at least one sample of a given species was considered as present in that species. These two datasets were analyzed in the same way: Jaccard dissimilarity index was computed, dendrograms were derived using neighbour joining (NJ) and unweighted pair group method with arithmetic mean (UPGMA) and plotted with `dendextend` package. Suitability of NJ vs UPGMA clustering methods was checked using plots of pairwise distances on a tree vs. original pairwise distances (Fig. 1 and 2). The both methods yielded comparable results for sample clustering; NJ performed slightly better for clustering of consensus species proteomes (Fig. 1 in the main text); both algorithms produced topologically similar trees; results of UPGMA clustering of consensus proteomes are on the Fig. 3. UPGMA clustering of the initial (not “consensus”) proteomes are on the Fig. 2 in the main text; the corresponding NJ clustering is on the Fig. 4.

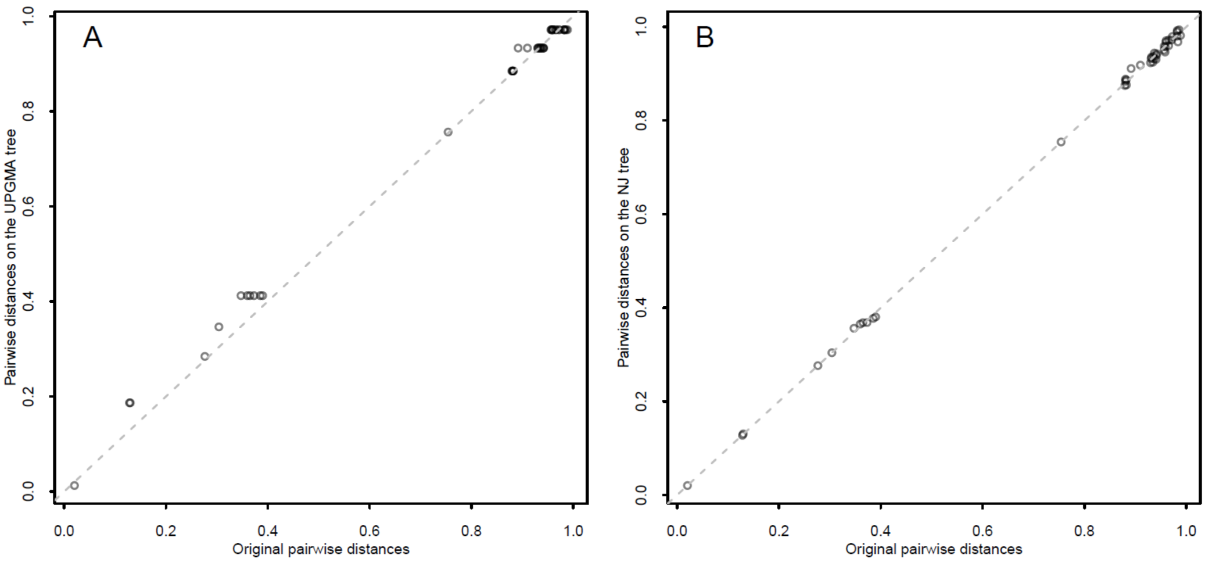


**Fig. 1. Choice of clustering method for consensus species proteomes.** Pairwise distances on a tree (either unweighted pair group method with arithmetic mean (A) or neighbour joining (B)) are plotted against original pairwise Jaccard dissimilarities of protein occurrence frequency in samples of different species. Dashed line marks the points where tree- and original distances are equal. Neighbour joining method performed slightly better for clustering of consensus species proteomes.

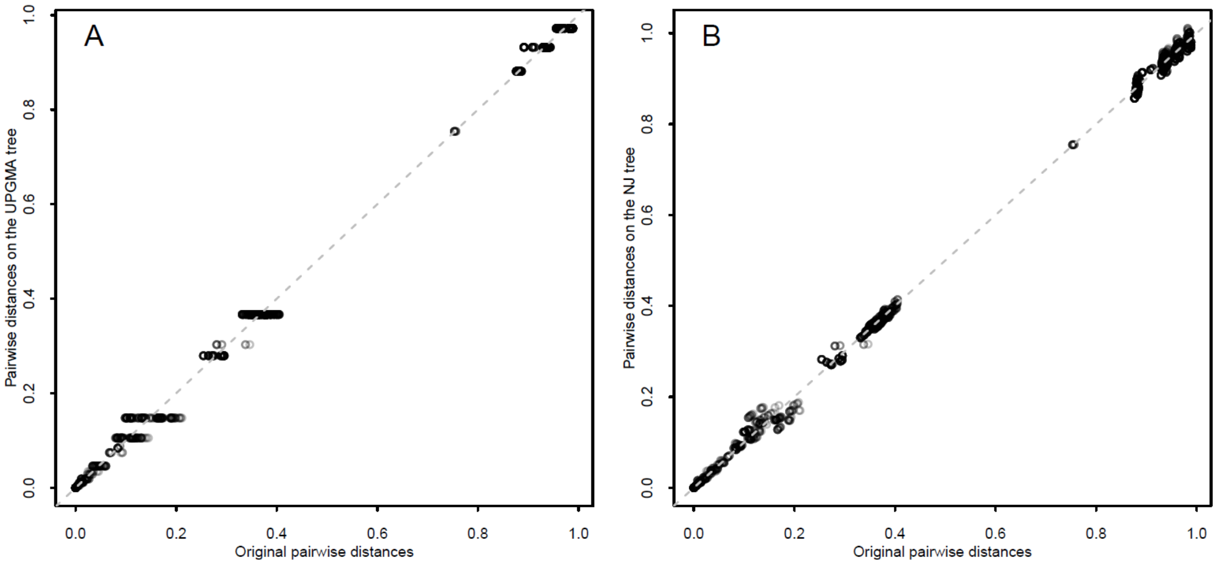


**Fig. 2. Choice of clustering method for proteomes in the samples.** Pairwise distances on a tree (either unweighted pair group method with arithmetic mean (A) or neighbour joining (B)) are plotted against original Jaccard dissimilarities of protein presence/absence in samples of different species. Dashed line marks the points where tree- and original distances are equal. The both clustering methods performed comparably well.

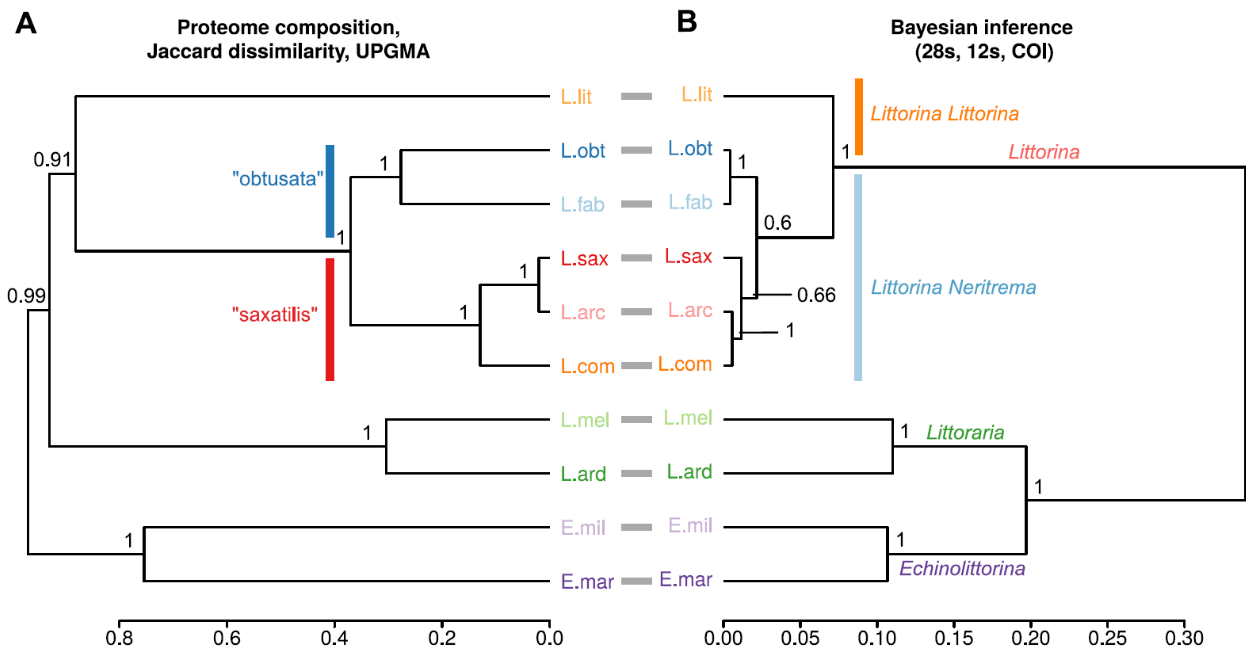


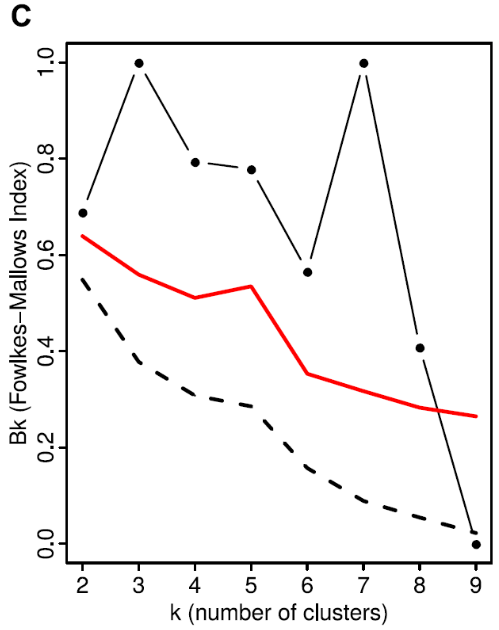


D

Proteomic (UPGMA-based) tree cophenetic distances.

| | E.mar | E.mil | L.ard | L.mel | L.com | L.arc | L.sax | L.fab | L.obt |
|---|---|---|---|---|---|---|---|---|---|
| **E.mil** | 0.777 | | | | | | | | |
| **L.ard** | 1 | 1 | | | | | | | |
| **L.mel** | 1 | 1 | 0.313 | | | | | | |
| **L.com** | 1 | 1 | 0.958 | 0.958 | | | | | |
| **L.arc** | 1 | 1 | 0.958 | 0.958 | 0.133 | | | | |
| **L.sax** | 1 | 1 | 0.958 | 0.958 | 0.133 | 0.021 | | | |
| **L.fab** | 1 | 1 | 0.958 | 0.958 | 0.381 | 0.381 | 0.381 | | |
| **L.obt** | 1 | 1 | 0.958 | 0.958 | 0.381 | 0.381 | 0.381 | 0.285 | |
| **L.lit** | 1 | 1 | 0.958 | 0.958 | 0.908 | 0.908 | 0.908 | 0.908 | 0.908 |

DNA-based tree cophenetic distances.

| | E.mar | E.mil | L.ard | L.mel | L.com | L.arc | L.sax | L.fab | L.obt |
|---|---|---|---|---|---|---|---|---|---|
| **E.mil** | 0.314 | | | | | | | | |
| **L.ard** | 0.580 | 0.580 | | | | | | | |
| **L.mel** | 0.580 | 0.580 | 0.324 | | | | | | |
| **L.com** | 1 | 1 | 1 | 1 | | | | | |
| **L.arc** | 1 | 1 | 1 | 1 | 0.017 | | | | |
| **L.sax** | 1 | 1 | 1 | 1 | 0.034 | 0.034 | | | |
| **L.fab** | 1 | 1 | 1 | 1 | 0.064 | 0.064 | 0.064 | | |
| **L.obt** | 1 | 1 | 1 | 1 | 0.064 | 0.064 | 0.064 | 0.013 | |
| **L.lit** | 1 | 1 | 1 | 1 | 0.210 | 0.210 | 0.210 | 0.210 | 0.210 |

**Fig. 3. Interspecies relations within the family Littorinidae.** (A) Dendrogram of consensus species proteomes obtained using unweighted pair group method with arithmetic mean (UPGMA) based on Jaccard dissimilarities of protein occurrence frequency in samples of different species. The bootstrap support values are shown. (B) The molecular phylogeny tree obtained via Bayesian inference using concatenated partial gene sequences from 28S rRNA, 12S rRNA and cytochrome oxidase C subunit I (COI). Support values are posterior probabilities. Prior to comparison, the molecular phylogeny tree was made ultrametric using non-negative least squares. Robinson-Foulds distance between unrooted trees was RF = 2 (normalized RF = 0.143). The cophenetic correlation between trees A and B is CC=0.844. (C) Fowlkes-Mallows index comparing dendrograms A and B. Black line with dots shows the change of the compositional similarity of clusters (Bk) with the number of clusters (k). Dashed line indicates Bk values under a null hypothesis of insignificant similarity of cluster' composition in the trees under comparison). Red line depicts threshold values for rejection of the null hypothesis. (D) Matrices of cophenetic distances for the proteomic and DNA-based trees expressed as a percentage of the total tree length.

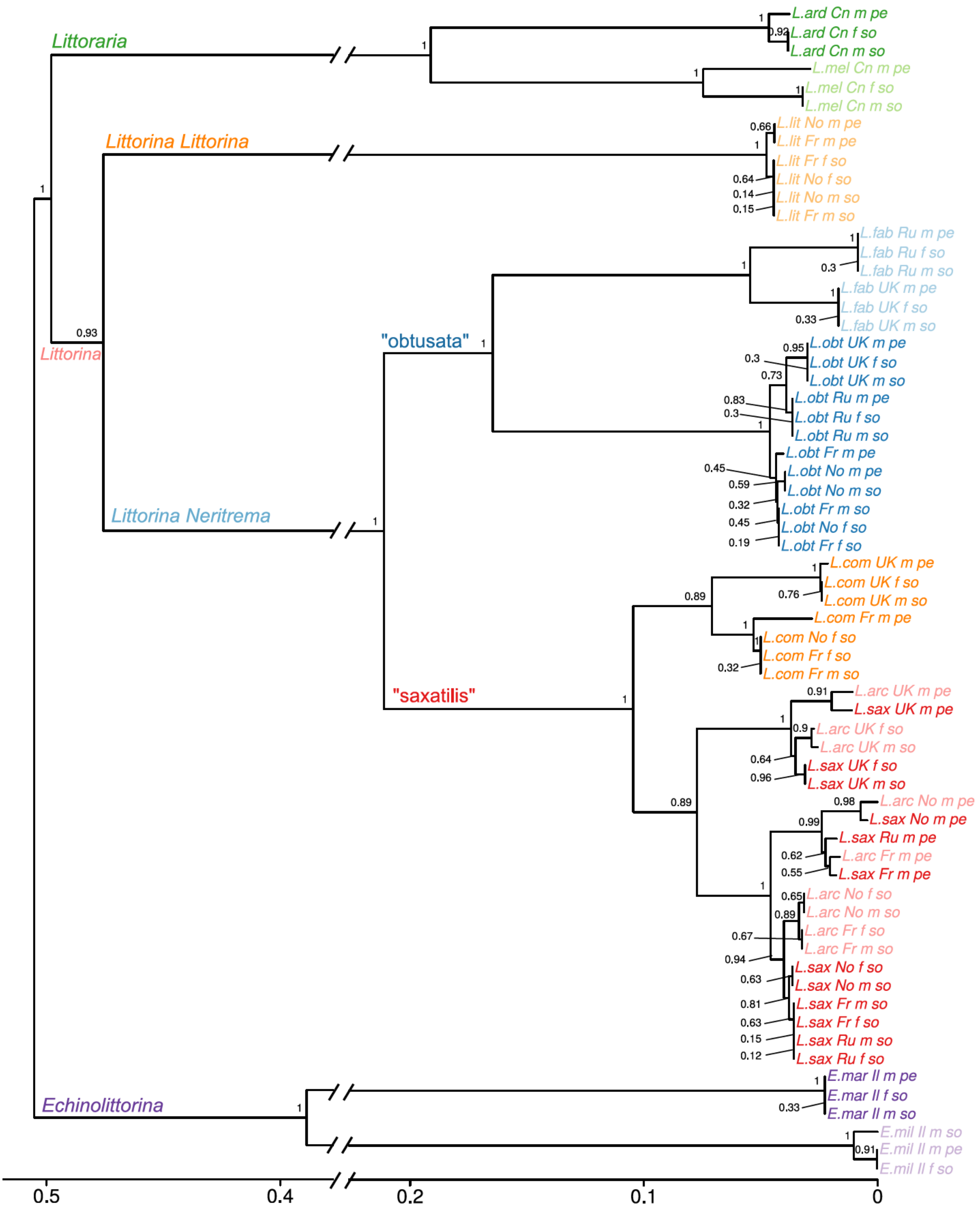


**Fig. 4. Dendrogram of proteomes from samples of the 10 Littorinidae species.** Clustering was performed using neighbour joining algorithm based on Jaccard dissimilarity coefficients for the data on presence/absence of proteins in the samples. Sample labels indicate species (L.arc: Littorina arcana; L.comp: L. compressa; L.sax: *L. saxatilis*; L.obt: *L. obtusata*; L.fab: *L. fabalis*; L.lit: *L. littorea*; L.ard: *Littoraria ardouiniana*; L.mel: *L. melanostoma*; E.mar: *Echinolittorina marisrubri*; E.mil: *E. millegrana*), location (Ru: White Sea, Russia; Fr: English Channel, France; UK: Atlantic coast, Scotland; No: Barents Sea, Norway; Cn: East-China Sea, Hong Kong; Il: Israel), sex (f: female; m: male) and body part (so: foot + head parts; pe: penis). The approximately unbiased bootstrap support values are shown.
